# Association of bullous pemphigoid and Grover disease induced by immune checkpoint therapy^☆^

**DOI:** 10.1016/j.abd.2023.07.018

**Published:** 2024-06-13

**Authors:** Elena Lucía Pinto-Pulido, Isabel Polo-Rodríguez, Marta González-Cañete, Ileana Medina-Expósito, María Dolores Vélez-Velázquez, Susana Medina-Montalvo

**Affiliations:** a Department of Dermatology, Hospital Universitario Príncipe de Asturias, Universidad de Alcalá, Madrid, Spain; b Department of Pathology, Hospital Universitario Príncipe de Asturias, Universidad de Alcalá, Madrid, Spain

Dear Editor,

Immune checkpoint inhibitors (ICIs) have recently been established as an essential therapeutic tool for several advanced cancers, including melanoma and other cutaneous malignancies. Immune-related adverse events (irAEs) are a frequent complication of these treatments, being the skin one of the most affected organs.^1^ Overall, a total of 25 %–50% of patients receiving ICIs (including CTLA-4, PD-1, and PD-L1 inhibitors) develop cutaneous irAEs.^2^, ^3^

We present a 90-year-old man treated with pembrolizumab (PD-1 inhibitor) because of an advanced earlobe squamous cell carcinoma (pT2pN2bM0). Two months after initiating pembrolizumab he complained about the presence of pruritic blisters and crusted erosions localized on neck and abdomen. One month later he noticed the appearance of other pruritic skin lesions on the back, with the persistence of blisters and erosions that now also affected his lower limbs. Skin examination showed erythematous papules of 3–5 mm distributed all over his back. In the right laterocervical area there was a large erythematous plaque with a serous blister and several crusted erosions. Other small crusted erosive lesions were observed on the hypogastrium, lower back, and both legs (Fig. 1).

**Fig. 1:**
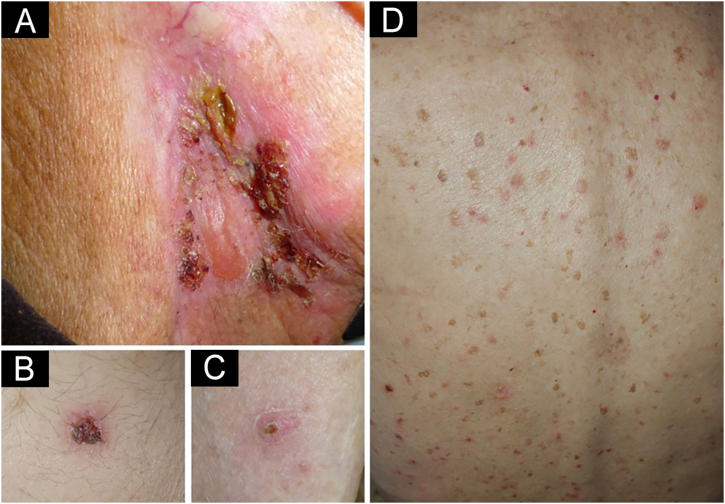
Erythematous plaque with a serous blister and crusted erosions in right laterocervical area (A). Crusted erosions localized in the lower abdomen (B) and pretibial area (C). Erythematous papules distributed over the patient's back (D).

Two 4 mm skin biopsies were obtained from the dorsolumbar area. Histology showed a parakeratotic hyperkeratosis with suprabasal acantholysis and dyskeratotic cells. The superficial dermis presented a lymphocytic inflammatory infiltrate with some eosinophils (Fig. 2A). Direct immunofluorescence (DIF) showed a strong linear C3 and IgG positivity along the basement membrane (Figs. 2B, 2C). Histological and clinical findings suggested the concurrence of Grover disease (GD) and bullous pemphigoid (BP). Despite clobetasol propionate topical treatment, skin lesions and itching worsened. Therefore, pembrolizumab was discontinued, and oral prednisone was started (tapered dose from 20 mg/day), resulting in skin lesions clearance.

**Fig. 2:**
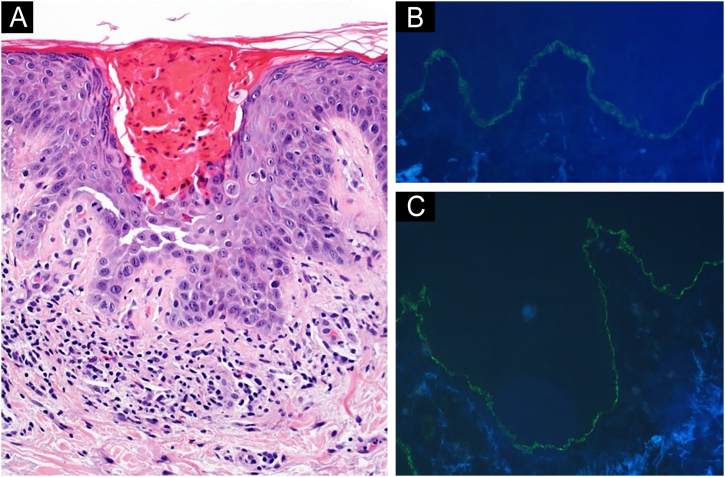
(A) Hematoxylin & eosin, ×20; of one of the erythematous papules on the patient's left dorsolumbar area: (A) Parakeratotic hyperkeratosis, suprabasal acantholysis and dyskeratotic cells are observed. A discrete lymphocytic inflammatory infiltrate with some eosinophils is noticed in superficial dermis. Direct immunofluorescence staining of normal left dorsolumbar skin, adjacent to a crusted erosion: linear IgG (B) and C3 (C) deposits are found along the basement membrane.

Among patients receiving ICIs, the reported incidence of BP is 0.3%–1%^1^, ^3^ and 0.2% for GD. Significant higher incidences were found with respect to a cohort of controls who did not use these therapies.^3^ The development of both diseases concomitantly in patients undergoing immunotherapy has so far been reported in three cases. All of them received nivolumab, another PD-1 inhibitor, and were men. Time to onset was 3, 9 and 18 months after ICI initiation. In contrast to our patients, they first developed features of GD and afterward of both GD and BP.^4^, ^5^

Concomitant GD and BP in patients not receiving ICIs have also been described. In all of them, GP preceded BP or appeared at the same time.^6^, ^7^ Therefore, one of the proposed hypotheses is that frequent scratching due to GD pruritus favors cell destruction and thus increases exposure to antigens such as BP320, altering the immune response and favoring BP development.^7^ However, this would not explain our patient's time sequence. Another hypothesis is that both diseases are triggered by the same factor.^7^ Despite not being able to prove causality with certainty, we consider that in our case the development of both entities was triggered by pembrolizumab, as it was observed 2–3 months after its initiation, it resolved after ICI withdrawal and both entities have been previously associated with PD-1 inhibitors (Naranjo Adverse Drug Reaction Probability Scale 4, possible). The concurrence of clinical and histological data of both diseases in our case and in those previously reported makes us suggest that the mechanisms by which this drug triggered both diseases may be related to each other.

When facing clinically and histologically atypical skin lesions, we must consider the possible concurrence of two different dermatoses. In patients with ICIs treatment, this possibility is greater since a higher incidence of several cutaneous diseases has been demonstrated.

## Financial support

None declared.

## Author’s contribution

Elena Lucía Pinto-Pulido: Data collection; literature review; effective participation in research orientation; preparation and writing of the manuscript.

Isabel Polo-Rodríguez: Data collection; Literature review; effective participation in research orientation; manuscript review; read and approved the final manuscript.

Marta González-Cañete: Literature review; effective participation in research orientation; helped in writing of the manuscript; read and approved the final manuscript.

Ileana Medina-Expósito: Data collection; Literature review; effective participation in research orientation; read and approved the final manuscript.

María Dolores Vélez-Velázquez: Data collection; literature review; effective participation in research orientation; read and approved the final manuscript.

Susana Medina-Montalvo: Data collection; literature review; effective participation in research orientation; manuscript review; read and approved the final manuscript.

## Conflicts of interest

None declared.
